# Neuroimaging Findings and Neurocognitive Features of Patients with Ochoa Syndrome (Urofacial Syndrome)—A Prospective Experimental Study

**DOI:** 10.3390/diagnostics15192488

**Published:** 2025-09-29

**Authors:** Aykut Akinci, Murat Can Karaburun, Mehmet Fatih Ozkaya, Muhammed Arif Ibis, Tugba Babayigit, Merve Cikili Uytun, Elif Peker, Sena Unal, Seda Kaynak Sahap, Gozde Vatansever, Sertac Ustun, Tarkan Soygur, Berk Burgu

**Affiliations:** 1 Department of Urology, Pamukkale University, 20070 Denizli, Turkey; 2 Department of Pediatric Urology, Ankara University, 06500 Ankara, Turkey; 3 Department of Urology, Etlik City Hospital, 06010 Ankara, Turkey; 4 Department of Urology, Ankara University, 06620 Ankara, Turkey; 5 Department of Pediatric and Adolescent Psychiatry, Ankara University, 06570 Ankara, Turkey; 6 Department of Radiology, Ankara University, 06570 Ankara, Turkey; 7 Department of Pediatric Radiology, Ankara University, 06570 Ankara, Turkey; 8 Department of Interdisciplinary Neuroscience, Ankara University, 06570 Ankara, Turkey; 9 Department of Physiology, Ankara University, 06100 Ankara, Turkey

**Keywords:** Ochoa syndrome, Urofacial syndrome, functional MRI, facial abnormality, lower urinary tract dysfunction

## Abstract

**Background/Objectives:** To characterize functional brain activation during smiling and to assess cognitive profiles in patients with Ochoa (Urofacial) syndrome (UFS). **Materials and Methods:** In a block-design fMRI paradigm, participants alternated between imitating a smiling emoji and viewing a fixation cross. Images were preprocessed and analyzed in SPM12; Smile > Rest contrasts were tested with a voxelwise threshold of *p* < 0.001 (uncorrected). Cognitive levels were assessed using age-appropriate Wechsler scales administered by certified psychologists. **Results:** Six patients (mean age 20 years; 50% female) with genetically/clinically confirmed UFS were included. Smile > Rest elicited robust activation in the supplementary motor area (highest Z = 4.70), insula (largest cluster), dorsal anterior cingulate, primary motor cortex, and frontal eye fields, among others. Five patients completed cognitive testing; Full-Scale IQ ranged 50–74, consistent with mild intellectual disability to borderline intellectual functioning. **Conclusions:** During voluntary smiling, UFS patients exhibit activation patterns that overlap extensively with those reported in healthy cohorts. Nevertheless, cognitive performance was limited in this sample. Given the rarity of UFS and the small cohort, findings should be interpreted cautiously and validated in multicenter studies.

## 1. Introduction

Urofacial syndrome (UFS), also known as Ochoa syndrome, is a very rare genetic disease with an autosomal recessive inheritance pattern. It is characterized by lower urinary tract dysfunction (LUTD) and facial abnormalities [1]. UFS patients experience difficulty in voiding due to sphincter–detrusor dyssynergia [1]. This causes high bladder pressures and increased post-void residuals, leading to vesicoureteral reflux, hydronephrosis, urinary tract infections, and renal scarring. All these conditions predispose patients to chronic kidney disease [2]. The inverted facial expression (appearing to cry while smiling) is pathognomonic for the disease [1].

To understand the underlying mechanism, Ochoa proposed the theory of central injury, based on the close proximity of the pontine micturition center and the facial nerve nucleus [2]. Another hypothesis has suggested peripheral neurological damage in the pathophysiology of UFS, proposing that the clinical findings may result from a peripheral neuropathy involving both the facial nerves and the nerves controlling micturition [3]. However, the pathophysiology of UFS has not been adequately elucidated. Moreover, no pathological findings have been detected in the brain and spinal cord MRI images of these patients [4].

Data regarding the neurocognitive functions of these patients remain limited. Although case reports have suggested that the intellectual functions of individuals with UFS are normal, no comprehensive studies using validated instruments to evaluate intelligence have been reported to date [5].

Given the limited literature in this field, we aimed to investigate the neuroimaging findings during laughter and to conduct a comprehensive assessment of the cognitive features of patients with Ochoa syndrome. To our knowledge, this is the first study to evaluate both the intellectual functions and the fMRI findings of patients with UFS.

## 2. Materials and Methods

### 2.1. Participants

This study was conducted in accordance with the ethical principles of the 1964 Declaration of Helsinki and its subsequent revisions, and was authorized by the local ethics committee (İ04-135-22). After approval by the institutional review board, six patients aged between 16 and 25 years were included in this study.

Inclusion criteria were as follows: patients had to have a clinically and genetically confirmed diagnosis of Ochoa syndrome, and possess sufficient physical and mental capacity to undergo the fMRI procedure, as well as provide written informed consent (or have consent obtained from their legal guardian). Exclusion criteria included any additional psychiatric or neurological disorders (e.g., epilepsy, anxiety disorder, depression); visual or hearing impairment severe enough to interfere with stimulus perception; contraindications for fMRI scanning such as metallic implants, pacemakers, or severe claustrophobia; and cognitive or behavioral problems that would prevent adequate compliance with the study protocol.

### 2.2. Experimental Design and Stimuli Presentation

**Figure 1:**
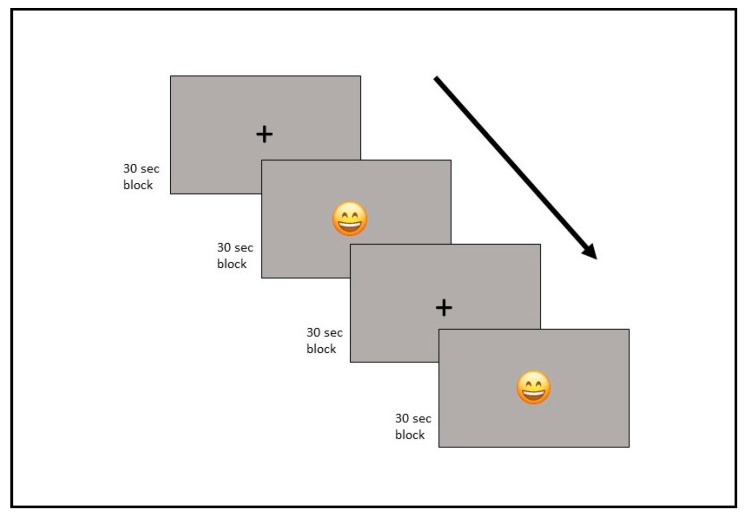
Block design task conditions.

A functional MRI experiment was performed using a block design consisting of active and resting conditions. The experiment employed two visual stimuli. Stimuli were presented at the center of the screen interchangeably for 30 s per block. The first block presented a smiling symbol (emoji), and the other presented a cross sign (resting) (Figure 1). Participants viewed the stimuli on a projection screen via a mirror attached to the head coil. They were instructed to imitate the smiling emoji with their facial muscles when the emoji appeared, and to rest when the cross sign appeared. Each block was repeated six times, with a total scan duration of 6 min.

### 2.3. fMRI Data Acquisition

Functional MRI images were acquired using a 3-Tesla MR scanner (Verio, Siemens, Erlangen, Germany) equipped with a 12-channel head coil. High-resolution T1-weighted anatomical images were obtained (TR: 1900 ms, TE: 2.52 ms, FOV: 250 × 250 mm, slice thickness: 1 mm, number of slices: 176). Gradient echo field mapping images were also acquired (TR: 488 ms, TE: 4.92 ms, FOV: 192 × 192 mm, slice thickness: 3 mm, number of slices: 36). Finally, BOLD echo-planar images (EPI) were collected (TR: 3000 ms, TE: 30 ms, FOV: 192 × 192 mm, slice thickness: 3 mm, number of slices: 36).

### 2.4. Image Preprocessing and Statistical Analysis

Imaging data were analyzed with SPM12 software (Wellcome Department of Cognitive Neurology, London, UK) running on MATLAB R2022a (The MathWorks Inc., Natick, MA, USA). Realignment was performed to correct for motion artifacts. To enable anatomical localization of activations, the realigned functional images were coregistered with the high-resolution T1-weighted anatomical images. Structural and functional images were then spatially normalized.

Following preprocessing, functional neuroimaging data were statistically analyzed using a paired t-test with the SPM12 factorial design feature. Smile > Rest contrasts are reported below. A voxel-level threshold of *p* < 0.001 was applied for significance. Results are reported with cluster size, laterality, MNI coordinates, and Z-scores. Cluster size indicates the degree of involvement within the activation region, with larger values reflecting greater activation. MNI coordinates specify the exact localization of the activation zone [6,7]. The Z-score reflects the level of statistical significance of regional activation, with higher values indicating stronger significance.

### 2.5. Cognitive Assessment

To assess cognitive skills, all patients underwent a DSM-5-based diagnostic psychiatric evaluation conducted by a child and adolescent psychiatrist. The Wechsler Intelligence Scale for Children–Revised (WISC–R) [8] (for children aged 6–16 years) or the Wechsler Adult Intelligence Scale–Revised (WAIS–R) [9] (over 16 years old) was administered by experienced, certified psychologists to determine patients’ cognitive levels. The obtained Full-Scale IQ scores were interpreted according to the Diagnostic and Statistical Manual of Mental Disorders, 5th Edition (DSM-5), which defines intellectual disability based on deficits in both intellectual and adaptive functioning [10]. Accordingly, the following IQ score ranges were used as guidelines for interpretation: 71–84 for borderline intellectual functioning, 50–55 to ~70 for mild intellectual disability, 35–40 to 50–55 for moderate intellectual disability, 20–25 to 35–40 for severe intellectual disability, and below 20–25 for profound intellectual disability.

## 3. Results

A total of six patients from five different families were included in the study. The mean age was 20 years, and three patients (50%) were female. Genetic analysis had been performed in all cases, and the diagnosis of Ochoa syndrome was previously confirmed.

All patients had been followed in the pediatric urology clinic for at least six years. They all presented with varying degrees of lower urinary tract dysfunction and an inverted facial expression. Review of urologic histories revealed that five patients had undergone subureteral injection procedures, three had ureteroneocystostomies, four had augmentation cystoplasties, and two had kidney transplantation for chronic renal failure. All patients were receiving anticholinergic medications for lower urinary tract dysfunction, and the two transplant recipients were maintained on standard immunosuppressive therapy protocols.

**Figure 2:**
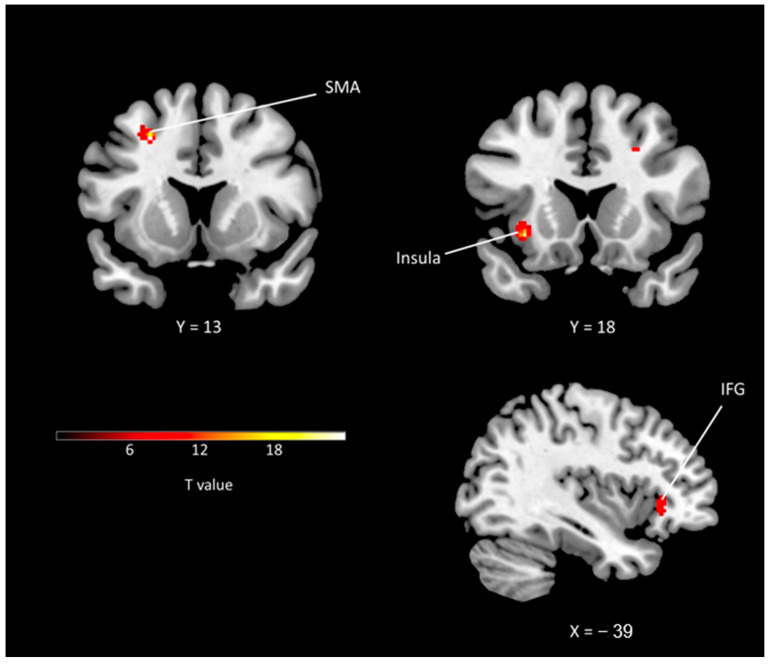
Activations revealed by smiling condition. IFG: inferior frontal gyrus; SMA: supplementary motor area.

The supplementary motor area demonstrated the highest Z-score (4.70) and the second largest cluster size (50). The insula showed the largest cluster size (113) and the second highest Z-score (4.63). Additionally, the dorsal anterior cingulate (Z-score: 4.51, cluster size: 50), primary motor area (Z-score: 4.50, cluster size: 25), and frontal eye field were among the other regions with significant activation. The anterior prefrontal cortex, cerebellum, inferior frontal cortex, sensory association cortex, thalamus, parahippocampal cortex, caudate, and ventral posterior cingulate were also activated during laughter (Table 1). Figure 2 illustrates the areas of brain activation and the degree of activation.

Cognitive levels were evaluated in five of the six patients. Verbal IQ scores ranged from 44 to 83, and Performance IQ scores ranged from 62 to 76. Full-Scale IQ scores were between 50 and 74 (Table 2). Verbal IQ, Performance IQ, and Full-Scale IQ scores of all patients fell within the ranges of mild intellectual disability or borderline intellectual functioning.

## 4. Discussion

In this study, we evaluated brain activity during laughter in patients with Ochoa syndrome using fMRI. In addition, we conducted a comprehensive diagnostic psychiatric evaluation and assessed their cognitive functioning. As both findings were obtained for the first time in this patient group, we believe that our study contributes to a better understanding of Ochoa syndrome.

In the current study, we found that the bilateral sensory and motor networks, including the primary and supplementary motor areas, sensory association cortex, and frontal eye field, were significantly activated during smiling. The insula, parahippocampal gyrus, inferior frontal cortex, anterior prefrontal cortex, and both the dorsal and ventral anterior cingulate were also involved in smiling. There appear to be two distinct neural pathways underlying the expression of laughter. The thalamic, hypothalamic, subthalamic, and dorsal tegmental brainstem regions are part of the first, the “involuntary” system. The premotor and frontal opercular regions form the starting point of the second, the “voluntary” system, which proceeds to the ventral brainstem via the motor cortex and pyramidal tract [11]. In our study model, we expected the second system, that is, the voluntary laughter pathway, to be activated. Since there was no control group, we could not compare our findings directly with healthy volunteers. However, our results can be compared with those of previous fMRI studies.

Considering the sensitivity of neuroimaging methods to movement, the neural processes underlying the expression of laughter remain unclear. Nevertheless, certain brain regions are known to show significant activation during laughter.

In a study with a similar design, Osaka and colleagues examined fMRI images during auditory command-induced laughter. In the experimental design, they obtained fMRI images of the subjects who were given laughter-inducing auditory stimulus and control auditory stimulus in blocks. The results of 14 healthy volunteers between the ages of 20 and 27, who were university students or university graduates included in the study, were examined. It was observed that extensive cortical areas including the left inferior frontal gyrus, left premotor (PM)/supplementary motor area (SMA) area, right anterior cingulate, left auditory area, right superior temporal gyrus, bilateral extrastriate visual cortex, and left hippocampus were activated during laughter [12]. The regions we found that are activated during laughter in Ochoa patients are similar to the regions activated in healthy volunteers shown by Osaka et al. Naturally, we can also see that different regions are activated due to differences in the experimental plan (auditory stimulation–visual stimulation).

Prior research has revealed that the supplementary motor area (SMA) is an important region for laughter [13]. Additionally, humorous cartoons and other stimuli that elicit laughter can modulate SMA activity [14]. Consistent with previous literature, we observed that the SMA was the region with the highest Z-score in our study. This finding suggests that patients with Ochoa syndrome exhibit brain activation patterns similar to those of healthy individuals. In another study by the same group, striatal reward centers including the putamen, caudate, nucleus accumbens, prefrontal cortices, dorsal anterior cingulate cortex, and SMA were activated during laughter compared with the control condition [15]. The superior frontal gyrus (Z-score: 4.74), anterior cingulate cortex (Z-score: 3.83), inferior frontal gyrus (Z-score: 3.47), middle frontal gyrus (Z-score: 3.46), and cerebellum (Z-score: 4.63) were the regions activated in the study by Osaka et al. The Z-scores obtained in their study were similar to those observed in patients with Ochoa syndrome in our cohort. Taken together with previous studies in healthy individuals, our findings suggest that the supplementary motor area and the anterior cingulate cortex, two critical regions for the motor control of laughter, are intact and functioning properly in patients with Ochoa syndrome.

However, laughter is such a complex expression that intact brain activations alone do not imply normal laughter. It requires intact neural impulses from the brain and properly functioning facial muscles. The inverted facial expression observed in patients with Ochoa syndrome may result from brain lesions, which we were unable to demonstrate, or from impaired neural transmission to, or dysfunction of, the facial muscles.

Comprehensive cognitive assessment of patients with Ochoa syndrome is lacking in the current literature. We performed such an assessment for the first time, to our knowledge. Since the IQ scores of all patients were consistent with mild intellectual disability or borderline intellectual functioning, this represents an important clinical finding for this rare genetic syndrome. These patients may experience chronic renal failure and consequent developmental delay as a result of lower urinary tract dysfunction. When accompanied by an underlying cognitive impairment, these problems may lead to difficulties in social functioning. Holistic evaluations may help clarify potential difficulties in social functioning and treatment adherence among children and adolescents with the syndrome and provide support when necessary. Therefore, we presume that comprehensive cognitive evaluations should be conducted in this specific patient group. Given the relatively small number of patients in this study, larger cohorts are needed to draw more definitive conclusions.

The major limitation of our study is the small sample size, which inevitably reduces the generalizability of the findings. This limitation is further compounded by the absence of a comparable control group, which would have allowed direct statistical comparisons with healthy individuals. However, Ochoa syndrome is an extremely rare autosomal recessive disorder, and it is inherently difficult to recruit larger patient populations or to design case control studies within a single center. In fact, most of the available literature consists of individual case reports or very small case series, highlighting the challenges of conducting large-scale research in this field. In this context, even small patient cohorts may yield meaningful insights, particularly when combined with advanced techniques such as functional neuroimaging and standardized cognitive testing. Despite these limitations, our study represents an important preliminary step in describing the neurocognitive and neuroimaging features of Ochoa syndrome. To address these challenges in the future, multicenter collaborations and the establishment of international patient registries will be essential to validate and build upon our findings.

## 5. Conclusions

In conclusion, our study demonstrated that brain activity elicited during facial expressions in patients with Ochoa syndrome demonstrated activation patterns similar to those observed in healthy individuals. At the same time, the intellectual functioning of this patient group was limited, with IQ scores consistent with mild intellectual disability or borderline intellectual functioning. These findings, although preliminary, provide important insights into the neurocognitive and neuroimaging features of this rare disorder.

Given the rarity of Ochoa syndrome, our results should be interpreted with caution and cannot be generalized to all patients. Nevertheless, we believe that the combination of functional MRI and standardized cognitive assessment in this study provides a valuable foundation for future research. To validate and expand upon these preliminary findings, larger multicenter studies and international patient registries are warranted.

## Figures and Tables

**Table 1:** Activations revealed by smiling condition (*p* < 0.001).

			MNI Coordinates			
Brain Region	Cluster Size	Laterality	X	Y	Z	Z-Score
Contrast: Smile > Rest						
Supplementary Motor Area	50	L	−26	12	44	4.70
	13	L	−42	6	40	3.32
Insula	113	L	−32	18	−8	4.63
Dorsal Anterior Cingulate	50	R	18	30	16	4.51
Primary Motor Area	25	R	12	−26	64	4.50
Frontal Eye Field	17	R	26	22	36	4.04
Anterior Prefrontal Cortex	12	L	−32	52	−8	3.89
	14	R	30	42	−6	3.74
	31	L	−24	40	0	3.70
Cerebellum	31	R	10	−66	−16	3.87
	13	R	4	−92	−18	3.70
Inferior Frontal Gyrus (pars opercularis)	26	L	−52	16	12	3.82
Sensory Association Cortex	20	L	−22	−28	40	3.79
Thalamus	29	L	−2	−22	12	3.55
	16	L	−20	−26	16	3.46
Parahippocampal Gyrus	56	R	4	−40	0	3.48
Caudate	32	R	20	−24	18	3.48
Ventral Posterior Cingulate	16	R	30	−54	10	3.37

**Table 2:** Cognitive levels of patients.

Patients	Verbal IQ Scores	Performance IQ Scores	Full-Scale IQ Scores
Patient 1	44	62	50 (mild intellectual disability)
Patient 2	58	76	65 (mild intellectual disability)
Patient 3	83	70	74 (borderline intellectual functioning)
Patient 4	58	62	57 (mild intellectual disability)
Patient 5	75	69	72 (borderline intellectual functioning)

## Data Availability

The data presented in this study are available on request from the corresponding author. They are not publicly available due to ethical considerations and protection of participant confidentiality.
